# Pathology Laboratory Archives: Conservation Quality of Nucleic Acids and Proteins for NSCLC Molecular Testing

**DOI:** 10.3390/jpm14040333

**Published:** 2024-03-22

**Authors:** Albino Eccher, Davide Seminati, Vincenzo L’Imperio, Gabriele Casati, Daniela Pilla, Umberto Malapelle, Isabella Piga, Greta Bindi, Alessandro Marando, Emanuela Bonoldi, Emanuele Dainese, Mattia Riefolo, Antonia D’Errico, Matteo Costantini, Alberto Lugli, Stefano Grassi, Aldo Scarpa, Angelo Paolo Dei Tos, Fabio Pagni

**Affiliations:** 1 Department of Diagnostics and Public Health, University of Modena and Reggio Emilia, 41121 Modena, Italy; albino.eccher@unimore.it; 2 Department of Medicine and Surgery, Pathology, Fondazione IRCCS San Gerardo dei Tintori, University of Milano-Bicocca, 20900 Monza, Italy; vincenzo.limperio@unimib.it (V.L.); g.casati8@campus.unimib.it (G.C.); daniela.pilla@irccs-sangerardo.it (D.P.); fabio.pagni@unimib.it (F.P.); 3 Department of Public Health, Pathology, University of Naples Federico II, 80131 Naples, Italy; umbertomalapelle@gmail.com; 4 Department of Biomedical Sciences, University of Cagliari, 09124 Cagliari, Italy; isabella.piga@unimib.it; 5 Proteomics and Metabolomics Unit, Department of Medicine and Surgery, University of Milano-Bicocca, 20900 Monza, Italy; g.bindi@campus.unimib.it; 6 Department of Pathology, ASST Grande Ospedale Niguarda, 20162 Milan, Italy; alessandro.marando@ospedaleniguarda.it (A.M.); emanuela.bonoldi@ospedaleniguarda.it (E.B.); 7 Department of Pathology, ASST Valtellina e Alto Lario, 23100 Sondrio, Italy; emanuele.dainese@asst-val.it; 8 Pathology Unit, Department of Diagnostic Medicine and Prevention, Sant’Orsola-Malpighi Hospital, University of Bologna, 40138 Bologna, Italy; mattia.riefolo@unibo.it (M.R.); antonietta.derrico@unibo.it (A.D.); 9 Department of Pathology, University Hospital of Modena, 41121 Modena, Italy; costantini.matteo@aou.mo.it (M.C.); lugli.alberto@aou.mo.it (A.L.); 10 Pathology Unit, IRCCS San Raffaele Scientific Institute, 20132 Milan, Italy; grassi.stefano@hsr.it; 11 Department of Diagnostics and Public Health, Section of Pathology, University and Hospital Trust of Verona, 37126 Verona, Italy; aldo.scarpa@univr.it; 12 Department of Pathology, University of Padua School of Medicine, Azienda Ospedale Università Padova, 35122 Padua, Italy; angelo.deitos@unipd.it

**Keywords:** FFPE blocks, pathology laboratory archive, conservation quality, NSCLC

## Abstract

In the molecular era, proper archival conditions within pathology laboratories are crucial, especially for formalin-fixed paraffin-embedded (FFPE) tissue specimens retrieved years after the original diagnosis. Indeed, improper preservation can impact the integrity of nucleic acids and protein antigens. This study evaluates the quality status of stored FFPE blocks using multilevel omics approaches. FFPE blocks from 45 Non-Small Cell Lung Carcinoma (NSCLC) cases were analyzed. The blocks were collected from six different pathology archives across Italy with distinct environmental characteristics. Nucleic acids’ quantity and quality, as well as protein antigens, were assessed using various techniques, including MALDI-MSI. RNA was quantitatively higher, but more fragmented, compared to DNA. DNA quantity and quality were suitable for molecular analyses in 94.4% and 62.3% of samples, respectively. RNA quantity was adequate across all samples, but it was optimal only in 22.3% of cases. DNA quality started to deteriorate after 6–8 years, whereas RNA quality diminished only after 10 years of storage. These data might suggest a particular DNA susceptibility to FFPE blocks conservation. Immunohistochemical intensity decreased significantly after 6–8 years of storage, and MALDI-MSI analysis revealed that younger tissue blocks contained more unique proteomic signals than the older ones. This study emphasizes the importance of proper FFPE archiving conditions for molecular analyses. Governance should prioritize attention to pathology archives to ensure quality preservation and optimize predictive testing. By elucidating the nuances of FFPE block storage, this research paves the way for enhanced molecular diagnostics and therapeutic insights regarding oncology and beyond.

## 1. Introduction

The correct maintenance of archives in pathology laboratories is compelling in the molecular era, especially for specimens retrieved years after an original diagnosis [1]. The detrimental impact of improper preservation of formalin-fixed paraffin-embedded (FFPE) tissue material, specifically related to temperature and humidity, might affect the quality of nucleic acids (leading to excessive fragmentation) and protein antigens (resulting in signal loss and antigenicity degradation) [2,3,4,5]. The National Cancer Institute’s Best Practices for Biospecimen Resources recommends preserving FFPE blocks with humidity ranging from 30% to 70% while maintaining a maximum temperature of 26 °C, or at least a maintained stable temperature that is never above or below 80/−20 °C [6,7,8,9]. Proper preservation depends on many other variables, including type of anesthesia, surgical procedure (manipulation and warm ischemia), transportation (cold ischemia), fixative type, fixation time and tissue exposure to dehydration, heat, UV radiation, urea, and salts [10]. On pre-cut archived blanks sections, nucleic acids and proteins have demonstrated a significant reduction when stored as early as 2 weeks after sectioning, in particular when exposed to direct light [11,12,13,14]. Moreover, previous studies have demonstrated that FFPE blocks are best suitable for Next Generation Sequencing (NGS) analysis—in terms of quality of library preparation and post-mapped results—within the first 3–7 years [15,16,17]. Furthermore, according to a multivariable analysis, DNA integrity emerges as the foremost independent factor associated with NGS success [17]. Starting from an Italian experience of institutes at different longitudinal and latitudinal sites, the current study applies multilevel omics approaches to test the quality status of stored FFPE blocks and evaluate the proper preservation of archived samples. Our comprehensive evaluation will focus on nucleic acid quantity (concentration) and quality (fragmentation index), as well as on protein antigens (immunohistochemistry—IHC—and matrix assisted laser desorption ionization mass spectrometry imaging—MALDI-MSI). The conclusions will enhance how the governance should pay prompt attention to pathology archives for an adequate standard of quality in the molecular medicine era to control the pre-analytical variable of NGS tests and the predictive relevance of results.

## 2. Materials and Methods

### 2.1. FFPE Blocks

**Figure 1:**
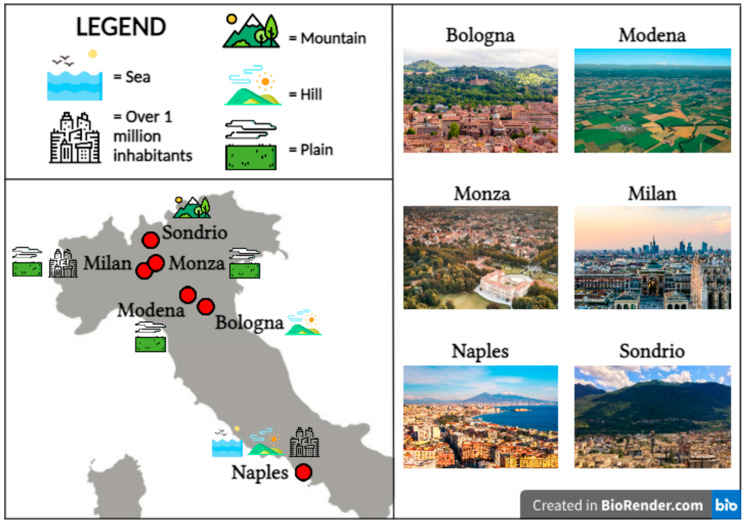
Geographical and environmental features of the six Italian centers.

FFPE left-over materials from surgical resection specimens of 45 Non Small Cell Lung Carcinoma (NSCLC) were used for the study, including both adenocarcinoma (ADC, 53%) and squamous cell carcinoma (SCC, 47%) histotypes. The former is the most often molecularly analyzed histotype in Italy, while the latter is the second most common malignant neoplasm in the thoracic district [18,19]. Cases were collected from six different Italian standard pathology archives located in geographical areas with distinct environmental characteristics (Table 1 and Figure 1).

Based on the current real world Italian scenario, specific formalin types, fixation times and storage conditions were largely unknown. The storage time ranged from 1 to 17 years (analyses performed in December 2023), with cases distributed as follows: 2006 (4 cases), 2007 (2), 2012 (9), 2015 (4), 2016 (2), 2017 (9), 2021 (7), 2022 (8) (Table S1). 

Cases were divided into three annual groups, each containing 15 samples, according to their storage time (Table 2):

Group #1: 2006–2012 with more than 10 years of storage.

Group #2: 2015–2017 with 6–8 years of storage (near the upper theoretical acceptability cutoff for NGS analysis of 7 years) [15].

Group #3: 2021–2022 with, at most, 2 years of storage (near the lower theoretical acceptability cutoff for NGS analysis of 3 years [17].

**Table 2:** Annual groups of samples according to their year of storage initiation.

Annual Group Progressive Number #	Number of Cases	Year of Storage Initiation	Storage Time (Years)
#1	15	2006–2012	>10
#2	15	2015–2017	6–8
#3	15	2021–2022	<3

### 2.2. Nucleic Acids Concentration and Fragmentation Testing

For each FFPE block, a slide stained with hematoxylin and eosin (H&E) and four unstained serial sections of 8 μm thickness were prepared.

**Figure 2:**
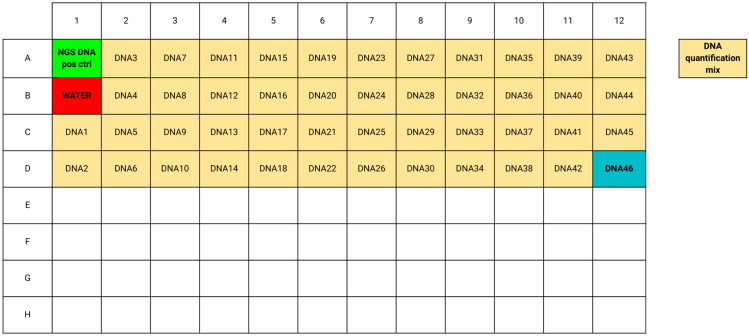
EasyPGX qPCR Instrument 96 sample grid with two positive controls (NGS DNA pos ctrl in green and DNA 46 in blue), a negative control (water, in red), and the 45 NSCLC DNA samplesfor the DNA fragmentation analysis.

Tumor cell enrichment was performed through manual macrodissection (scraping the unstained FFPE tissue slides with a sterilized scalpel into a sterile eppendorf tube) only on areas previously circled with a black marker on H&E slides by a pathologist (DS), carefully avoiding necrosis or improper fixation areas. The automatic extraction and quantification of nucleic acids (DNA and RNA) was carried out using the Ion Torrent™ Genexus™ Purification System (GPI, Thermo Fisher Scientific, Waltham, MA, USA) with the Ion Torrent™ Genexus™ FFPE Combo Kit. The fragmentation index testing was conducted using the Myriapod NGS Cancer panel DNA/RNA fragmentation assay with the real-time PCR EasyPGX qPCR instrument 96 (Diatech Pharmacogenetics, Jesi, Italy). This assay enables the detection of two highly conserved genomic regions measuring 127–184 bp and 105–175 bp for DNA and RNA, respectively. Detection was achieved through probes labeled with FAM (shorter amplicons) and HEX (longer amplicons). The fragmentation index was analyzed using the EasyPGX Analysis Software version 4.0.13, and it was calculated as the ratio between the concentration in ng/μL obtained in the HEX channel and that determined in the FAM channel. The analysis was conducted starting from a concentration of 1 ng/μL (within the method’s specified range of 0.0625–8 ng/μL) for both DNA and RNA (after reverse transcription to cDNA) using a volume of 25 μL. The analysis also included positive and negative (water) controls provided by the assay itself, as well as one positive control with known fragmentation index obtained by the routine diagnostics of center #4 (Figure 2).

The samples were considered suboptimal for molecular analysis in the presence of a nucleic acid concentration < 5 ng/μL and/or a low fragmentation index, as per the manufacturer’s instructions (Table 3) [20,21].

### 2.3. Protein Antigens Analysis: IHC and MALDI-MSI Approaches

For each H&E slide, a tumor cell rich area was circled by a pathologist (DS) within the larger region previously selected for nucleic acid extraction. This area was used to craft a FFPE tissue microarray (TMA, tissue cores with diameter of 1 mm) for conducting IHC and proteomic analyses. At the conclusion, 45 tissue cores were present, but only 44 could be correctly assessed, as the core from Sample 3 could not be included in the analyses due to its partial laceration during the TMA preparation phase. Six one-micron-thick TMA sections were cut and mounted onto TOMO Adhesion Microscope Slides (Matsunami Glass, Japan) for IHC. Three nuclear (TTF1—clone SP141, p40—clone BC28, MIB1—clone 30-9), two membrane (EMA—clone E29, CD34—clone QBend10), and one membrane/cytoplasmic stainings (anti-Pan Keratin—clone AE1/AE3/PCK26) were performed on a fully automated BenchMark ULTRA platform (Ventana Medical Systems, Tucson, AZ, USA). The proper preservation of IHC staining in tissue cores was assessed based on an intensity score using a four-level semiquantitative ascending scale (0, 1, 2, 3) by two pathologists (DS, FP) (Table 4).

One five-micron-thick TMA section was cut and mounted onto conductive indium tin oxide (ITO) glass for MALDI-MSI analysis, as previously described [22]. The MALDI-MSI analysis was performed using a rapifleX MALDI Tissuetyper (Bruker Daltonics GmbH, Bremen, Germany) MALDI-TOF/TOF MS equipped with a Smartbeam 3D laser operating at 5 kHz frequency. Mass spectra were acquired in the reflectron positive mode, within the m/z 700−3000. The MALDI–MS images were acquired with a single-spot laser setting of 100 μm and a scan range of 96 × 96 μm. A mixture of standard peptides within the mass range of m/z 750 to 3150 (PepMix I, Bruker Daltonics, Bremen, Germany) was used for the external calibration and directly applied on the glass slide (mass accuracy < 10 ppm). FlexControl 4.2 (Bruker Daltonics, Bremen, Germany) was used to set up the instrument parameters for the acquisition method, while FlexImaging 6.0 (Bruker Daltonics, Bremen, Germany) was utilized for the MALDI–MSI analysis visualization. The data files containing the individual spectra of the entire TMA was imported into SCiLS Lab v.2023a Pro software (Bremen, Germany), and standard pre-processing of the data was performed, including baseline subtraction (TopHat algorithm), normalization (Total Ion Current algorithm), weak spatial denoising, alignment and peak picking. The average (avg) spectra, representative of each core, were generated in order to display the differences in the proteomic profiles. A total of 191 m/z features were detected within the entire dataset. The maximum peak intensity of each of the 191 m/z signals was imported in Metaboanalyst 6.0 and exploratory unsupervised multivariate analysis using heatmaps with hierarchical clustering analysis (HCA) was performed.

## 3. Statistical Analysis

Data analysis was performed using Excel software v.2108 (Microsoft, Redmond, WA, USA). A Student’s *t*-test was employed for quantitative variables and a Fisher’s exact test was used for categorical variables. To examine the relationships between some variables, we employed the Pearson correlation coefficient (r). All *p* values were two-sided and a < 0.05 level was considered to be statistically significant. A proteomic statistical analysis and exploratory data analysis were conducted using Metaboanalyst 6.0 [23]. The open-source software mMass v.5.5 (http://www.mmass.org accessed on 27 December 2023) was used for further analysis and mass spectra visualization [24].

## 4. Results

### 4.1. Nucleic Acids Raw Data Results

The cohort’s average, median, maximum and minimum DNA/RNA concentrations and fragmentation index are summarized in Table 5 (all detailed data are shown in Table S1).

Overall, 77.7% (35/45) cases were suboptimal for molecular analysis based on at least one nucleic acid quantity/quality value below the threshold (Table 6).

All these cases had a low RNA quality but a good RNA quantity. DNA was below the cutoff for quantity or quality in 6.6% (3/45) and 37.7% (17/45) of the samples, respectively, 3 with both.

As biologically expected, RNA was quantitatively higher compared to DNA (*p* < 0.001), but it was more fragmented (*p* < 0.001). No significant differences were found comparing ADC and SCC histotypes for DNA and RNA.

### 4.2. Annual Groups Evaluation

**Figure 3:**
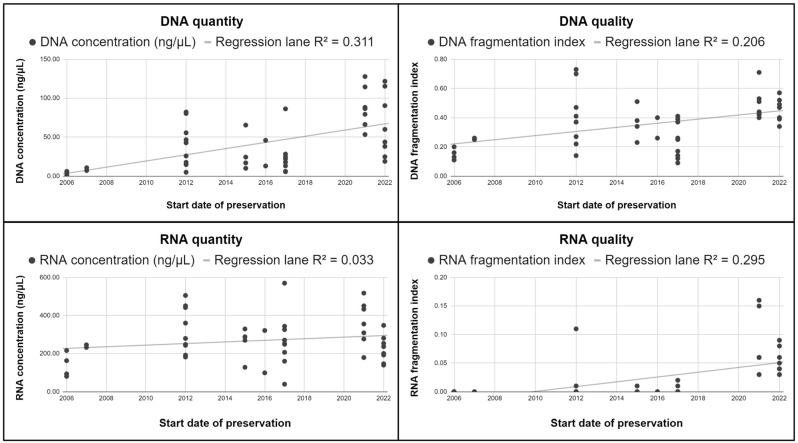
Association between start date of preservation and nucleic acids quantity and quality as for linear regression.

Among the annual groups, DNA quantity and RNA quality showed a significant decrease from 6–8 years of storage (group #2, *p* = 0.005 and *p* < 0.001, respectively) without further differences comparing older samples (groups #1 vs. #2). No differences were found among the annual groups in terms of DNA quality and RNA quantity. Linear regression lanes are shown in Figure 3.

Comparing each annual group among the various centers, we found higher DNA concentrations in the most recent samples of center #4 (*p* = 0.048), lower DNA concentrations in the oldest samples of center #1 (*p* = 0.011) and lower RNA fragmentations in groups #2 and #3 of center #4 (*p* = 0.001 and *p* = 0.022, respectively) and group #1 of center #6 (*p* = 0.009). When comparing centers based on environmental characteristics, the only significant difference found was a higher RNA quantity in the archives located in the plain areas (*p* = 0.038).

### 4.3. Nucleic Acids Results According to Molecular Adequacy Criteria

Performing the same comparative analyses based on adequacy criteria for molecular analysis, RNA showed a significantly higher fragmentation only in the oldest cases (*p* = 0.005), while DNA started to be too fragmented from 6–8 years (*p* = 0.002). Moreover, center #1 oldest samples of DNA and more recent samples of RNA proved to be more fragmented compared to all other centers (*p* = 0.027 and *p* = 0.041, respectively). Comparison of centers by environmental features showed no significant differences.

### 4.4. IHC Results

IHC intensity score data are shown in Table 7.

**Figure 4:**
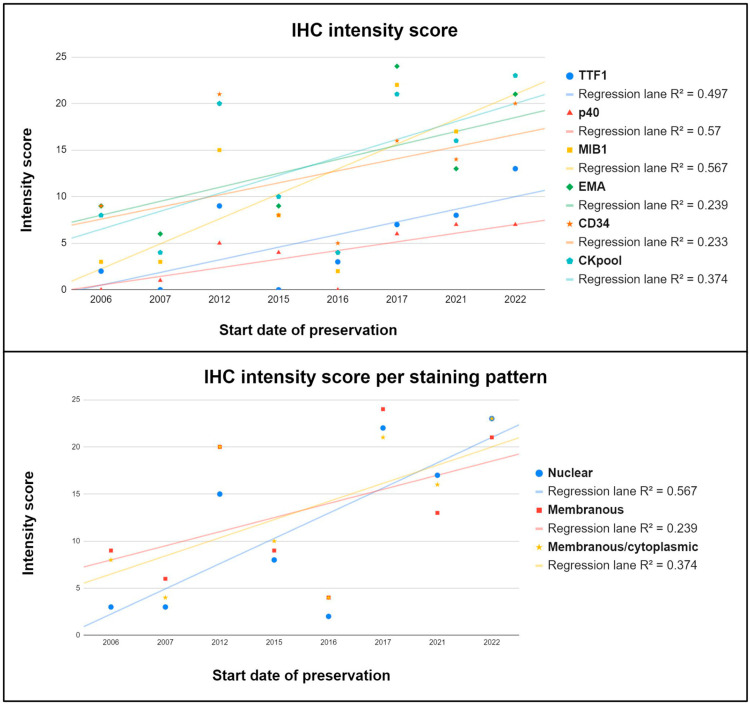
Association between immunohistochemical (IHC) intensity score with start date of preservation as for linear regression.

Comparing the IHC intensity scores between the annual groups, a significant decrease was observed both after 6–8 years and >10 years of storage (*p* = 0.007 and *p* < 0.001, respectively). Comparison of archive centers by annual groups or by environmental features showed no significant differences. Linear regression lanes are shown in Figure 4 (TTF1 and p40 lower intensity scores depend mainly on histotype and the variable positivity of these stains).

Stratifying IHC stains by staining pattern (considering only MIB1 staining for the nuclear category for the above mentioned limitations of p40 and TTF1), we found a particularly significant decrease in nuclear staining intensity for samples stored for >10 years (*p* < 0.001). Comparing IHC expression overall with nucleic acid data, we found only weak correlations. However, restricting the attention only to the nuclear antigen MIB1, the level of correlation increased (*p* value passed from *p* = 0.001 to *p* < 0.001).

### 4.5. MALDI-MSI Proteomic Results

**Figure 5:**
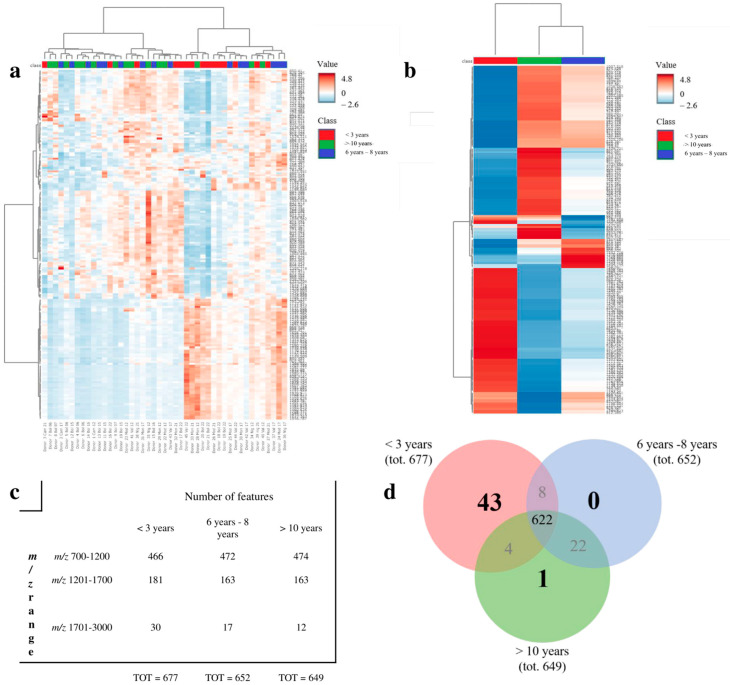
Exploratory analysis of the proteomic profiles of FFPE samples archived for different periods of time obtained from MALDI-MSI analysis. Hierarchical cluster analysis (HCA) with heatmap visualization showing (**a**) individual FFPE samples and (**b**) the group averages for each time of sample conservation. Green: Group #1 (>10 years); Blue: Group #2 (6–8 years); Red: Group #3 (<3 years). Heatmaps were created with MetaboAnalyst 6.0. The data were autoscaled; Euclidean was used as a distance measure and Ward as a clustering method. (**c**) Table containing the total number of m/z features detected in the average mass spectra of group #1, #2 and #3 in the following mass spectra regions: m/z 700–1200, m/z 1202–1700, and m/z 1701–3000, with S/N ≥ 6 and an intensity threshold of 0.5%. (**d**) Venn diagram comparing the number of common features between the average mass spectra of group #1, #2 and #3 cases.

The proteomic analysis with MALDI revealed distinct clustering patterns (Figure 5a), with two major nodes corresponding to cases stored for >10 years (group #1, green) and <3 years (group #3, red), as well as group #2 cases (6–8 years) distributed within the older ones (blue). This was further confirmed by the heatmap based on averaged values per group (Figure 5b), which highlighted signal intensities shared by group #1 and #2 with most recent cases (group #3), showing a distinct expression pattern. When observing the number of features per each average spectra of the three sample groups, only cases with <3 years of storage (group #3) reached the adequacy criteria of signal-to-noise (S/N) ratio ≥6 and an intensity threshold of 0.5%, showing 4% more signals as compared to the others. Considering average mass spectra, the highest and lowest number of features were seen within the 700–1200 and 1701–3000 m/z range, respectively, with group #3 cases (<3 years) having >43% signals in this latter m/z range (Figure 5c). The three groups shared 622 features overall, with an average of 43 unique features for group #3 cases (<3 years) and 1 or none features extracted from group #2 (6–8 years) and #1 (>10 years), respectively (Figure 5d).

## 5. Discussion

Formalin-fixation and paraffin-embedding are the 100 year old routine methods for preserving tissue specimens in pathology laboratories due to their ability to maintain cellular structures over extended periods. It is estimated that more than a billion tissue samples—mainly FFPE—are currently archived in hospitals and biobanks around the world [25,26]. It is widely known that improper storage of FFPE blocks in archives affects their overall integrity and usefulness for subsequent analyses [3]. Recently, the emergence of advanced molecular investigations has rekindled interest in archived FFPE biological material [6]. Therefore, optimizing the storage conditions of FFPE specimens becomes crucial in ensuring the sustained integrity of these materials for analyses conducted years after the original diagnosis. While it is true that all biological materials undergo senescence from the moment they are collected, it is also important to acknowledge the potential long-term benefits of preserving samples and striving for better preservation methods. These can be particularly valuable for different reasons, starting from the heterogeneity of legislation and recommendations within different countries for the potentially prospective need to retrieve old samples for additional molecular analysis in the routine clinical setting, which encounter the creation and standardization of the biobanks for both clinical and research purposes. In this study, we assessed the preservation capability of six different standard pathology archives located in various regions of Italy characterized by distinct orographic and environmental features. The evaluation focused on both the quantity (concentration) and quality (fragmentation) of nucleic acids (DNA and RNA), as well as on the preservation of protein antigens using IHC and MALDI-MSI approaches. We placed particular emphasis on the rate of suboptimal cases for molecular analysis, following cutoff values verified and routinely utilized in the literature [20,21]. As biologically expected, RNA was quantitatively higher, but more fragmented, compared to DNA. Overall, an appropriate DNA quantity for molecular analysis was found in 94.4% of the samples, in line with data present in the literature, and an appropriate DNA quality was found in 62.3% of the cases due to a significantly increased fragmentation after 6–8 years of storage [12]. The RNA quantity was found to be optimal across the entire set of cases, while RNA quality was adequate only in 22.3% of specimens, with particularly poor data in the older samples and with a few isolated and limited exceptions, indicating a general difficulty in the management of this nucleic acid. RNA reached an absolute level of fragmentation—no longer measurable within the limits of the technology employed (real-time PCR)—after 11 years of storage. In summary, based on the raw data, the FFPE blocks currently archived in Italy demonstrate effective preservation of DNA quality and RNA quantity exclusively, while there is a noteworthy decline in both DNA quantity and RNA quality after 6–8 years of storage. However, when applying adequacy criteria for molecular testing, the sole preservation of DNA and RNA quantities appeared satisfactory. Paradoxically, DNA quality started to deteriorate after 6–8 years, whereas RNA quality diminished only after 10 years of storage. These data might suggest that, if RNA is known for its sensitivity to pre-analytical variables in specimen fixation, then DNA could be more susceptible to variability in FFPE block conservation. Curiously, we found higher RNA quantity in the archives located in plain areas (Milan, Modena, Monza); this is an interesting finding that may warrant further investigation through protein quantitative analysis, even if it may be affected by our relatively limited sample size. Comparing the IHC intensity scores between the annual groups, a significant decrease was observed after 6–8 years of storage, primarily due to the greater fragility of nuclear staining, particularly for MIB1. The pronounced degradation of this nuclear staining over time and the generally of stainings undergoing heat antigen retrieval is already known, and the antigenicity can be partially recovered by deep sectioning and lengthening of heat pretreatment [14,27]. In line with prior research, our MALDI-MSI proteomic analysis underscored that the duration of archival time does not impair protein profiling in FFPE tissues [28]. In fact, the number of common features among the three annual groups unveiled 622 (>90%) features shared across all categories. This finding emphasizes that FFPE samples still hold valuable and comparable proteomic information irrespective of archival time. On the other hand, our results highlighted that samples archived for fewer years contained a greater number of unique signals, with 43 signals in the proteomic profile that were not detected in the other two classes, while only 0 and 1 unique signals were detected in the groups 6–8 years and older than 10 years, respectively. These results suggest that younger tissue blocks harbor proteomic profiles richer in information compared to older blocks, thus potentially unveiling proteomic insights that might remain undetected when utilizing older tissue blocks. In conclusion, acknowledging the resilience of archival samples in retaining genomic and proteomic information, while recognizing the temporal nuances in signal diversity, may facilitate the FFPE block preservation management. This study focused mainly on the archival conditions of FFPE blocks in pathology laboratories, which are mainly affected by heterogeneity in terms of temperature and humidity. Although alternative preservation methods (e.g., Optimal Cutting Temperature compound, OCT) exist, they are less frequently used in clinical settings due to the complexity of the sample management (fresh-frozen specimens at −20/−30 °C temperatures) and storage (cryopreservation at ultra-low −80 °C temperatures or in liquid nitrogen) [29]. Even with the presence of similar nucleic acids and protein retrieval capacities as compared to the FFPE samples, the complex archiving requirements of these specimens imply a more strict control of environmental conditions, suggesting a minor impact of them on the final analyses [10].

The study has some limitations. First, it is characterized by a retrospective design. Second, it relies on a limited cohort. Lastly, it only considers a FFPE framework, while specific formalin types, fixation times and storage conditions among centers were largely unknown. Notwithstanding this, our research introduces innovative aspects, notably the inclusion of a geographic analysis based on environmental features and the integration of a combined multiomic examination of proteins and nucleic acids.

## 6. Conclusions

Based on this pivotal feasibility test, the importance of proper conditions for FFPE archiving may have significant consequences in the molecular pathology lab. The conclusions confirmed that the governance should pay prompt attention to pathology archives for an adequate standard of quality in order to control the pre-analytical variables of NGS testing and the predictive relevance of results. Moreover, a multilevel approach including the proteomic landscape of FFPE tissues showed how traceability and quality control of archives may increase the possible information that pathologists can obtain from their diagnostic and predictive tests.

## Figures and Tables

**Table 1:** Locations of the archives and FFPE blocks features. m. a.s.l.: meters above sea level; FFPE: formalin fixed, paraffin embedded.

Center Progressive Number #	Center	Geographic Coordinates	Altitude (m a.s.l.)	Range of Start Year of Storage	N° of FFPE Blocks
#1	Bologna	44°29′38″ N 11°20′34″ E	54	2006–2022	18
#2	Milan	45°28′01″ N 9°11′24″ E	120	2012–2021	6
#3	Modena	44°38′52″ N 10°55′31″ E	34	2012–2021	6
#4	Monza	45°35′01″ N 9°16′25″ E	162	2012–2021	6
#5	Naples	40°51’46″80 N 14°14′47″ E	17	2012–2021	3
#6	Sondrio	46°10′06″ N9°52′16″ E	360	2012–2022	6

**Table 3:** Fragmentation index cutoff values with their associated increasing level of fragmentation.

DNA Fragmentation Index	RNA Fragmentation Index	Nucleic Acid Fragmentation Level
>0.5	>0.7	Low
0.3–0.5	0.05–0.7	Medium
<0.3	<0.05	High: suboptimal for molecular analysis

**Table 4:** Fragmentation index cutoff values with their associated increasing level of fragmentation.

	Staining Intensity			
	Absent	Barely Perceptible/Weak	Moderate	Strong
IHC intensity score	0	1	2	3

**Table 5:** Cohort’s nucleic acid concentration (ng/μL) and fragmentation index.

	Average		Median		Minimum		Maximum	
Nucleic Acid	DNA	RNA	DNA	RNA	DNA	RNA	DNA	RNA
Concentration	42.68	269.16	25.83	252.41	1.46	39.27	127.84	569.94
Fragmentation index	0.36	0.025	0.38	0	0.09	0	0.73	0.16

**Table 6:** Cohort’s suboptimal rates according to DNA/RNA quantity and quality.

	Quantity		Quality	
Nucleic Acid	DNA	RNA	DNA	RNA
Suboptimal rate	6.6%	0%	37.7%	77.7%

**Table 7:** Immunohistochemical intensity score across annual groups.

	IHC Intensity Score		
Annual Group Progressive Number #	Sum	Median	Average
#1	139	2	1.54
#2	153	2	1.7
#3	182	3	2.17

## Data Availability

Data available on request from the corresponding author due to privacy restrictions.

## Supplementary Materials

The following supporting information can be downloaded at: https://www.mdpi.com/article/10.3390/jpm14040333/s1, Table S1: Complete data on sample center, storage date, DNA and RNA quantity and quality.
