# Supplementary material for: Pathology Laboratory Archives: Conservation Quality of Nucleic Acids and Proteins for NSCLC Molecular Testing
Source: J Pers Med. 2024 Mar 22;14(4):333. doi: 10.3390/jpm14040333 (PMC11050929; doi:10.3390/jpm14040333)
Supplement: Supplementary file 1 [file jpm-14-00333-s001.zip › Table S1.pdf]

Case number	Center	Start date of preservation	Histotype	DNA concentration (ng/µL)	DNA fragmentation index	RNA concentration (ng/µL)	RNA fragmentation index
1	BOLOGNA	2006	ADC	6,03	0,20	216,25	0,00
2	BOLOGNA	2006	ADC	5,88	0,11	163,03	0,00
3	BOLOGNA	2006	SCC	1,46	0,13	80,38	0,00
4	BOLOGNA	2006	SCC	4,33	0,16	93,33	0,00
5	BOLOGNA	2007	ADC	7,10	0,25	232,85	0,00
6	BOLOGNA	2007	SCC	10,68	0,26	246,00	0,00
7	BOLOGNA	2015	ADC	24,34	0,38	288,44	0,01
8	BOLOGNA	2015	SCC	16,89	0,23	269,85	0,00
9	BOLOGNA	2015	SCC	65,44	0,34	329,38	0,00
10	BOLOGNA	2015	SCC	9,97	0,51	128,10	0,00
11	BOLOGNA	2016	ADC	45,90	0,40	321,46	0,00
12	BOLOGNA	2016	ADC	12,99	0,26	98,92	0,00
13	BOLOGNA	2022	ADC	24,80	0,40	139,71	0,05
14	BOLOGNA	2022	ADC	43,72	0,52	192,61	0,04
15	BOLOGNA	2022	SCC	90,46	0,47	253,92	0,03
16	BOLOGNA	2022	SCC	115,39	0,57	348,35	0,08
17	BOLOGNA	2022	SCC	59,95	0,49	200,75	0,04
18	BOLOGNA	2022	ADC	121,75	0,47	281,01	0,03
19	MILAN	2012	ADC	25,83	0,70	182,17	0,00
20	MILAN	2012	SCC	4,90	0,14	279,44	0,00
21	MILAN	2017	ADC	86,32	0,41	252,41	0,00
22	MILAN	2017	SCC	28,25	0,26	569,94	0,00
23	MILAN	2021	ADC	66,30	0,40	433,16	0,03
24	MILAN	2021	SCC	88,23	0,51	355,65	0,03
25	MODENA	2012	ADC	82,21	0,47	442,01	0,00
26	MODENA	2012	SCC	42,73	0,37	505,55	0,00
27	MODENA	2017	SCC	17,80	0,14	343,62	0,00
28	MODENA	2017	ADC	5,86	0,17	160,08	0,00

29	MODENA	2021	SCC	53,34	0,43	179,47	0,06
30	MODENA	2021	ADC	79,34	0,42	517,81	0,06
31	MONZA	2012	ADC	55,58	0,27	451,27	0,00
32	MONZA	2012	SCC	17,64	0,37	191,82	0,01
33	MONZA	2017	ADC	24,63	0,39	326,16	0,01
34	MONZA	2017	SCC	22,20	0,37	270,50	0,02
35	MONZA	2021	ADC	127,84	0,71	276,68	0,16
36	MONZA	2021	SCC	114,52	0,53	450,78	0,15
37	NAPLES	2012	ADC	14,76	0,22	243,64	0,00
38	NAPLES	2017	ADC	6,10	0,12	248,88	0,00
39	NAPLES	2021	ADC	86,57	0,44	309,28	0,06
40	SONDRIO	2012	ADC	46,64	0,41	248,12	0,00
41	SONDRIO	2012	SCC	80,38	0,73	360,23	0,11
42	SONDRIO	2017	ADC	5,54	0,25	39,27	0,00
43	SONDRIO	2017	SCC	13,10	0,09	206,63	0,00
44	SONDRIO	2022	ADC	18,87	0,34	146,41	0,09
45	SONDRIO	2022	SCC	38,02	0,39	236,76	0,06
